# Pesantren and inclusion: Bridging religion and disability in Islamic education in Indonesia

**DOI:** 10.4102/ajod.v14i0.1741

**Published:** 2025-09-25

**Authors:** Nurul H. Rofiah, Norimune Kawai, Dara Sudiraharja

**Affiliations:** 1 The Institute for Diversity and Inclusion, Hiroshima University, Higashi-Hiroshima, Japan; 2 Center of Disability Studies and Service, Ahmad Dahlan University, Yogyakarta, Indonesia; 3 Department of Islamic Education, UIN Sunan Kalijaga, Yogyakarta, Indonesia

## Introduction

Pesantrens are a cornerstone of Islamic education in Indonesia, functioning as residential learning institutions where students [*santri*] live and study under the guidance of religious scholars [*kyai*]. Characterised by distinctive features such as communal living, intensive study of classical Islamic texts [*kitab kuning*] and an emphasis on moral and spiritual formation, pesantrens have been established for multiple generations and remain deeply embedded in the country’s educational landscape (Iqbal & Akram 2020; Islam & Aziz 2020; Hefner 2021; Kadir & Umiarso 2023). In recent decades, pesantrens have undergone significant transformations, expanding beyond their traditional role to incorporate formal and vocational education, community outreach and social development initiatives. They have increasingly played a crucial role in advancing inclusive Islamic education by adapting curricula, facilities and teaching methods to accommodate students from diverse ethnic, cultural, social and ability backgrounds (Afrianty 2019; Yusak, Madrah & Ardi 2023). These adaptations reflect the broader influence of pesantrens on Indonesian Muslim communities, shaping both religious identity and societal values (Ali 2011; van Wichelen 2010). As a result, pesantrens today are not only religious institutions but also dynamic centres of learning that bridge faith, education and inclusion (Nilan 2009; Pohl 2006). While pesantrens have long served as religious and moral education centres, their evolving role in accommodating persons with disabilities (PWD) marks a significant step towards inclusive Islamic education. This connection between pesantrens and disability inclusion is central to this article, ensuring that the discussion integrates both religious tradition and the rights of PWD from the outset.

Islamic education is a significant component of Indonesian society because it is responsible for shaping the character and religious values of individuals and offering both fundamental and advanced educational guidance using an Islamic framework (Chanifah et al. 2021). Pesantrens, as Islamic educational institutions, have a history dating back thousands of years in Indonesia (Suyatno et al. 2022). They offer comprehensive education (Anshori & Pohl 2022). Furthermore, Pesantrens in Indonesia’s multicultural setting contribute to preserving Islamic traditions and principles that have been developed for generations (Chaerunisa, Rizkia & Firdaus 2019; Mujahid 2021). Pesantrens accomplish this by incorporating native cultural elements into their Islamic teaching methods and offering education concerning the core principles of Islam, memorisation of the Quran [*tahfiz*], interpretation of the Quran [*tafsir*], Hadith, Islamic history and other aspects of the religion (Ma’Arif 2018; Syukur 2019).

Additionally, pesantrens also prioritise the cultivation of their pupils’ character and morals through the instruction of Islamic principles, such as integrity, self-control, accountability and empathy (Sulhan & Hakim 2023). Pesantrens promote social inclusivity by admitting students from many origins, including those who are impoverished, and contribute to the national education system by providing instruction in secular disciplines, such as social sciences, natural sciences and languages (Latif & Hafid 2021). Pesantren institutions actively engage in social initiatives and community empowerment, fostering student involvement in community and public services and local economic growth (Saifulloh 2021). This contributes to promoting social inclusion and sustainable development.

However, it is worth mentioning that there are various types of pesantrens with different approaches and areas of emphasis. Certain pesantrens adhere more strictly to traditional practices, whereas others are more receptive to educational advancement or are modern pesantrens (Hadi 2022). Nevertheless, pesantrens continue to be a significant component of the Islamic education system in Indonesia, generally fostering inclusion and religious principles.

In 1997, Indonesia enacted its first legislation to safeguard the rights of PWD – ensuring equal access to education, employment, healthcare and public facilities, as well as protection from discrimination – and to outline the obligations of relevant stakeholders to uphold these rights (Afrianty 2020; Ediyanto et al. 2019; Nurhayati 2020). This legislation, which facilitates the implementation of inclusive education, consists of *Law No. 4 of 1997* on PWD. However, this law was invalidated and replaced by *Law No. 8 of 2016* and *Law No. 19 of 2011*, which ratified the Convention on the Rights of PWD (Rofiah & Suhendri 2023). Numerous parties, including pesantren institutions, began actively supporting these concerns, because many individuals with disabilities expressed a keen desire to pursue studies at these institutions. Additionally, the latest regulation, the *Minister of Religious Affairs Regulation Number 1 of 2024*, concerning Adequate Accommodation for Students with Disabilities in Educational Institutions under the Ministry of Religious Affairs, strengthens these efforts. Article 2, paragraph 2 of this regulation mandates that the Minister provide adequate accommodation for students with disabilities in madrasahs, religious higher education institutions, religious education units and pesantrens.

This article examines pesantren’s contribution to fostering inclusive Islamic education in Indonesia. It explores their historical background, place within the national education system and guiding principles, as well as the role of the *kyai* in shaping inclusive practices. The article further analyses opportunities for education for all, including PWD, focusing on religious participation, adequate accommodations in education and worship and modifications in learning methods.

## Method

This study’s research method is a narrative literature review. It summarises existing literature and offers a rich and nuanced interpretation. This approach allows the researcher to develop a comprehensive understanding of the role of pesantren in supporting inclusive education for PWD. This method was chosen to gather, analyse and synthesise relevant material on religion and disability in Islamic education in Indonesia. The narrative literature review enables a thorough study of current literature while also considering the historical, social and cultural contexts that shape inclusive education in pesantren. This method was selected for its adaptability to different forms of literature and its capacity to review a complex topic comprehensively.

The literature gathering procedure involved searching multiple academic databases, such as Google Scholar, JSTOR and university libraries, to identify pertinent sources. The study utilised keywords such as ‘inclusive education in Islamic boarding schools’, ‘disability in Islamic education’ and ‘Pesantren practices in inclusivity’ to locate relevant publications, books and research reports. The selected literature explicitly examines the concept of inclusion in Islamic education, particularly in pesantren, and explores the correlation between religion and disability. The literature analysis was conducted by identifying prominent themes that emerged from the diverse sources. The material was classified according to themes.

## Discussion

### Pesantren in the Indonesian education system

Pondok, Dayah, Kobong, Surau, Meunasah and other similar terms – collectively known as pesantren – are communal establishments founded by individuals, foundations, Islamic community organisations or the community itself (Isbah 2016). Pesantrens aim to foster devotion and reverence towards Allah Swt., develop virtuous behaviour and uphold the teachings of Islam as a source of compassion for all beings [*rahmatan lil’alamin*] (Masrukhin & Supaat 2018; Na’imah & Nurdin 2017). The cultivation of humility, tolerance, balance, moderation and other noble values in Indonesia reflects this vision (Mashuri, Futaqi & Sulhan 2024). These objectives are achieved through education, Islamic preaching [*da’wah*], role modelling and community empowerment within the Indonesian context.

Pesantren education refers to the educational system implemented in these specialised Islamic boarding schools (Lathifah, Setyaningsih & Wulandari 2022). Conducted within the pesantren environment, the curriculum is tailored to the institution’s unique character (Ekaningrum et al. 2018), primarily based on traditional Islamic texts [*kitab kuning*] and focused on Islamic studies [*dirasah Islamiah*]. The educational model revolves around the *muallimin* [Islamic teachers] who guide students in their learning. *Santri* are students who pursue religious knowledge in pesantrens (Karim et al. 2022), while *kiai* – also referred to as Tuan Guru, Anre Gurutta, Inyiak, Syekh, Ajengan, Buya, Nyai and other regional titles – are respected Islamic scholars who serve as educators, role models and custodians of pesantren traditions (Sari & Dawud Faza 2024; Mukri & Tamam 2021).

As educators, *kiai* play a central role in preserving pesantren culture and distinctiveness (Dian et al. 2024; Yusuf & Taufiq 2020). This cultural identity includes nurturing Islamic principles such as benevolence towards all beings [*rahmatan lil’alamin*], tolerance, balance and moderation, anchored in nationalism and guided by Pancasila and the 1945 Constitution of the Republic of Indonesia. Within each pesantren, the *kiai* holds authority over institutional governance, including educational policies, curriculum direction and community rules – resulting in variations in policies and practices across pesantrens (Dakir, Fauzi & Anwar 2020).

In Indonesia’s national education system, pesantrens play a crucial role in providing Islamic education (Sulhan & Hakim 2023). Their distinctive attributes include a curriculum rooted in classical Islamic literature, the use of Arabic as a medium of instruction and pedagogical approaches that prioritise character and moral formation (Yaqin, Rozi & Sham 2020). According to the Ministry of Religious Affairs, there are approximately 22 115 pesantrens in Indonesia, with over 3 778 083 *santri* and 412 720 teachers (*kiai* or *ustadz*) (Indonesian Ministry of Religious Affairs 2022). Historically, pesantrens have functioned not only as educational institutions but also as centres for *da’wah* and community empowerment. In recognition of these three core functions, the Ministry promotes the policy slogan ‘Preserving Tradition, Guarding Innovation’.

Over time, many pesantrens have integrated non-religious subjects such as mathematics, English and science into their curricula (Azizah, Muchtar & Putra 2022; Isbah 2020). Some also operate government-recognised formal education programmes, including vocational and senior high schools (Maulida & Ali 2023; Yusuf & Taufiq 2020). For many Indonesians, pesantrens provide an alternative for deeper, specialised religious instruction (A’la & Rahman 2022) and offer educational access in rural or underserved areas (Al Idrus, Herlina & Ibrahim 2023).

Acknowledging their importance, the Indonesian government has introduced initiatives such as the ‘empowered pesantren’ programme to improve their educational standards (Hudaefi & Heryani 2019). To remain relevant in the modern era, pesantrens must continue to innovate while preserving their cultural and religious heritage, ensuring that they meet both contemporary educational demands and societal needs. These distinctive characteristics not only preserve pesantren traditions but also create opportunities to adapt their practices to meet the needs of students with disabilities, making them potential leaders in advancing disability-inclusive education in Indonesia.

### Principles of pesantren

Pesantrens are established based on core concepts that serve as the conceptual basis and approach for educating and fostering students. Geertz (1976) coined the term ‘santri’ to designate a socio-cultural group within the Muslim community, contrasting it with other groups such as ‘abangan’ and ‘priyayi’.

Geertz identifies two primary distinctions between the santri and abangan factions (Nashir & Jinan 2018). Firstly, they differ in their adherence to the fundamental teachings of Islam as outlined in the Quran and Hadith. Secondly, they diverge in perspectives on social structures and their respective roles in society. The Santri group prioritises the everyday practice of Islamic teachings as its core principle, while the Abangan group places greater emphasis on the traditional traditions of their community with less regard for religious ideas.

Currently, the term santri is used to denote individuals who engage in religious studies within pesantren institutions (Halid et al. 2024; Iqbal 2023). The primary tenet of pesantrens is to enhance the comprehension of Islamic doctrines and mould pupils into pious individuals. In pesantrens, teaching follows a conventional approach that relies on classical Islamic sources. In addition to enhancing Islamic teachings, pesantrens cultivate moral values and character in students through everyday education and practical encounters (Akmaliyah et al. 2021; Azra et al. 2007). Their lifestyles and activities demonstrate a strong emphasis on discipline and high independence, as seen in their ordered and structured approach.

Pesantrens also facilitate the development of pupils’ social skills in diverse settings, instructing them to value diversity and engage in cooperative efforts (Nawas, Darmawan & Maadad 2024). Additionally, pesantrens offer cost-effective education to the community, typically at modest prices and often with subsidies (Duncan 2020). They provide educational opportunities for communities in rural areas as substitutes for conventional schools, delivering easily accessible religious education to people from all social strata (Maunah 2009).

Pesantren education offers explicit guidance in moulding pupils into individuals with virtuous character, religious instruction and constructive societal contributions (Budiharso, Bakri & Sujito 2023). Indonesian pesantrens possess various characteristics that differentiate them from other institutions. Madrasas are traditional Islamic educational institutions that focus on teaching classical Islamic books using Arabic as the language of instruction and employing a teaching technique that prioritises character and moral development (Akmaliyah et al. 2021). Furthermore, the milieu of pesantrens is highly Islamic, characterised by prevalent religious practices such as collective prayers, the *adhan* [call to prayer] and adherence to Islamic dress codes. Additionally, pesantren institutions feature a robust religious curriculum that consistently prioritises the study of foundational Islamic writings, such as the Quran, Hadith, Fiqh and Tafsir.

Pesantrens foster moral values and character development through everyday education and practical encounters, imparting lessons on proper conduct, discipline and the importance of respecting others (Sulhan & Hakim 2023). Students must demonstrate a strong sense of self-reliance and self-control, as seen in their well-organised and disciplined ways of life and pursuits. Pesantrens foster the acquisition of social skills in diverse settings, instruct children to value diversity and collaborate effectively in groups (Maunah 2009). By embedding these principles within their educational philosophy, pesantrens can ensure that inclusivity – especially for PWD – is a natural extension of their core values.

### Role of a *kyai* in pesantrens

A *kyai* is an educator with expertise in Islamic religious knowledge who serves as a figure, role model and caretaker of pesantrens (Futaqi & Mashuri 2022). *Kyais* are highly respected by students, the community and the pesantren environment (Kurniawan et al. 2022). The presence of a *kyai* in the pesantren is crucial to its success in producing a generation of young people who are faithful, morally upright and prepared to face future challenges (Arif 2016; Kastamin et al. 2021).

To manage a pesantren, a *kyai* must meet certain qualifications, as stipulated in *The Law of the Republic of Indonesia Number 18 of 2019* concerning Pesantren Article 5, Paragraph 2, Letter [a]. These qualifications include pesantren education, higher Islamic religious education and expertise in Islamic knowledge. As the highest leader of pesantrens, *kyais* serve as a caretaker, figures and role models in the administration. In carrying out his duties, a *kyai* can be assisted by educators and educational staff with competencies according to the needs of the pesantren and pesantren administrators who support the *kyai*’s role in administrative management.

A *kyai* functions as a teacher responsible for instructing students in Islamic teachings and other knowledge, such as Arabic, the Quran, Hadith, Fiqh, Tafsir and Islamic history, as well as for instilling Islamic moral and ethical values (Roslan Mohd Nor & Malim 2014). *Kyais* also serves as spiritual leaders, guiding students on matters related to Islamic teaching, helping them deal with spiritual issues and providing advice and motivation (Khoirunnisa & Atabik 2024; Yaqin et al. 2020).

Additionally, *kyais* act as a social guide, assisting students in developing positive attitudes such as independence, discipline and responsibility and helping address social issues both within and outside pesantrens (Islam & Aziz 2020). *Kyais* also preserve pesantren traditions by maintaining and upholding long-established values and cultures while developing and adapting the pesantren to modern advancements (Sakai & Isbah 2014). Finally, *kyais* serve as the leader and manager of pesantrens and are responsible for running them effectively and efficiently, organising educational activities and fostering cooperation with the surrounding community to support inclusive environment (Stewart-Ginsburg et al. 2020). The *kyai*’s authority and leadership are therefore critical not only for maintaining religious traditions but also for initiating and sustaining inclusive practices that welcome and support students with disabilities.

### Pesantren and disability inclusion

Pesantrens have great potential to support the government in expanding access to education for all Indonesian children, including those with disabilities, through deliberate disability inclusion initiatives. These initiatives go beyond simply allowing enrolment; they involve adapting teaching methods, curricula, facilities and social attitudes so that students with disabilities can participate fully in both learning and community life. Most pesantrens in Indonesia offer education free of charge or at a very affordable cost, enabling children from underprivileged families – and particularly those with disabilities, who often face additional barriers – to pursue their studies without significant financial burden (Rohman & Muhtamiroh 2022; Subaidi et al. 2023). In addition to widening access, pesantrens implement character-based education and moral values that align with the government’s goal of building strong character and moral integrity among the younger generation while also nurturing empathy, respect and inclusion for all learners.

Beyond religious education, pesantrens equip students with practical skills such as writing, reading and speaking in Arabic and English (Pohl 2006; Rohman, 2022). These competencies enhance future opportunities for all students, including PWD, by enabling them to participate more fully in society and the workforce. Government support through programmes such as the Pesantren Development Program and Operational Education Assistance has further strengthened pesantren capacity, ensuring that quality education is more widely available, even in rural and underserved areas.

Partnerships between pesantrens and formal schools can further expand inclusive educational opportunities (Budiharso et al. 2023). Collaboration like student-teacher exchanges, joint training, and curriculum sharing – can help pesantrens adopt more inclusive teaching practices that respond to the diverse needs of learners. With sustained cooperation between pesantrens and the government (Kosim 2015; Safiudin, Qurtubi & Masfu’ah 2023), pesantrens can transform into fully inclusive educational institutions that welcome and support students with disabilities.

Recognising these opportunities, pesantrens can make a range of contributions that directly support the inclusion and empowerment of students with disabilities. Socially, pesantrens can foster inclusive interactions by integrating students with disabilities into all aspects of daily life, reducing stigma and building a strong sense of belonging through shared living and communal activities. Psychologically, a supportive pesantren environment can help strengthen confidence, resilience and self-esteem among students with disabilities, supported by the moral and spiritual guidance of *kyais* and teachers as well as encouragement from peers.

Educationally, pesantrens can adapt curricula, teaching strategies and assessment methods to meet diverse learning needs, employing differentiated instruction, assistive technology and flexible learning schedules so that students with disabilities can achieve their learning goals. Physically, they can provide accessible infrastructure such as ramps, disability-friendly toilets, safe transportation and appropriate classroom layouts, as well as assistive devices to support participation in all activities. Spiritually, pesantrens can ensure full participation in religious practices by providing braille Qurans, sign language interpretation and physical assistance for ablution and prayer, enabling students with disabilities to engage meaningfully in the spiritual life of the institution.

To implement these contributions effectively, pesantrens can invest in training and professional development for teachers and administrators to strengthen their capacity in inclusive education. Collaborations with social work organisations that focus on disability empowerment can further enhance resources and support systems (Maftuhin 2023). Additionally, designing specialised curricula tailored to the needs and characteristics of students with disabilities will ensure that their education is both relevant and meaningful.

By fostering inclusive values and modelling equitable practices for the wider community, pesantrens can raise awareness of the importance of disability inclusion in education. In doing so, they not only fulfil their traditional role as centres of faith and learning but also contribute significantly to the Indonesian government’s vision of inclusive education that provides equitable access for all children.

### Worship for people with disabilities in pesantren

Worship is a central element of life in pesantrens, and ensuring that PWD can participate fully in religious activities is a key component of disability inclusion. Pesantrens must provide access and opportunities for people with disabilities to worship appropriately and without obstacles. One important step is to provide adequate accessibility facilities, such as ramps, lifts and toilets, that people with disabilities can use (Badawy, Jawabrah & Jarada 2020; Petersen & Piletic 2006). These facilities are essential for ensuring that all students, regardless of their physical condition, can easily and comfortably access various areas of the pesantren (Amin, Zuki & Akhir 2019).

Additionally, pesantrens should provide explanations and guidance to students, especially those who memorise the Quran, so that they can perform worship well and understand the meaning of each verse they read (Kosim 2015; Sauri, Nursyamsiah & Nurbayan 2018). Detailed explanations and directed guidance will help PWD to feel more confident about their worship.

It is also crucial for pesantrens to have adequate facilities and infrastructure to support people with disabilities in performing worship (Nakua et al. 2017). These include wheelchairs, braille Qurans and audio Qurans. These resources will greatly assist people with disabilities in participating in worship activities independently and comfortably.

Moreover, the active involvement of pesantren staff and administrators in providing support and assistance to people with disabilities who wish to worship is essential (Rusmini et al. 2023). This support can include helping them reach the place of worship, preparing the necessary equipment and ensuring they can worship without obstacles.

Pesantrens also need to teach and guide other students in supporting and assisting people with disabilities in performing worship, such as helping to carry and assist people with disabilities in performing ablutions or prayers. This guidance builds student empathy and cooperation and creates a more inclusive environment.

In addition to physical support, pesantrens must provide psychological and social support to people with disabilities (Shikarpurya & Singh 2021). This includes helping them overcome their lack of confidence or self-esteem in performing worship. Such support can be provided through counselling, mentoring and specially designed motivational programmes. With comprehensive support, pesantrens can become inclusive institutions that provide opportunities for everyone, including people with disabilities, to worship without obstacles.

For example, pesantrens can provide seating for persons with mobility disabilities, ensuring that they can easily perform ablution (Riwanto et al. 2023). These steps can ensure that all the students, regardless of their physical condition, can perform worship with full devotion and without difficulty. By combining accessible facilities, inclusive worship resources and a culture of mutual support, pesantrens can ensure that spiritual life is accessible to all, strengthening their role as inclusive religious institutions.

### Adequate accommodations and learning modifications in pesantren

Providing adequate accommodations and learning modifications is essential for achieving disability inclusion in pesantrens. This means ensuring that PWD have equitable access to both educational and religious activities, free from physical, social and instructional barriers. As part of an inclusive approach, pesantrens must go beyond basic compliance and intentionally design their environments, curricula and teaching practices to support the diverse needs of all learners (Riwanto et al. 2023).

Physical accessibility is the foundation of inclusion. Pesantrens can provide disability-friendly facilities such as ramps, lifts, accessible toilets and dedicated ablution areas for PWD, ensuring that they can move freely and comfortably throughout the campus (Aji, Suhardi & Iftadi 2022; Lestari & Raodah 2020). For students with visual impairments, braille Qurans and tactile learning materials are crucial (Taufik et al. 2024), while environmental modifications such as adjustable seating, sufficient lighting and contrasting colours can improve orientation and comfort for all (Amandhila et al. 2023; Wright et al. 2019). These modifications not only assist people with disabilities but also provide comfort for all users.

Technology is another valuable tool for helping people with disabilities to access education and worship (Riwanto et al. 2023). Using braille devices, hearing aids and screen reader software enables students to learn and worship more effectively and independently. This helps people with disabilities overcome the barriers they may face in learning and worship.

The use of sign language is a crucial step in creating an inclusive environment (Murray, De Meulder & Le Maire 2018). Providing sign language interpretation to persons with hearing disabilities ensures that they can follow lessons and worship activities without communication barriers. This respects and facilitates their communication needs. By providing appropriate accommodations, everyone, including those with special needs, can fully access and benefit from education and worship without barriers (Sango & Forrester-Jones 2017).

These accommodations and modifications do not only benefit PWD; they enhance the learning environment for all students by fostering empathy, collaboration and respect for diversity. In line with the national education goals and the role of pesantrens as institutions that embody *rahmatan lil’alamin* [a mercy to all worlds] values, such inclusive practices strengthen pesantrens’ contribution to building an equitable education system (Maksum, Asy’arie & Aly 2020). By embedding these measures into their daily operations, pesantrens can position themselves as leaders in advancing disability-inclusive education in Indonesia.

## Conclusion

Pesantrens are key institutions in advancing inclusive Islamic education in Indonesia, increasingly bridging the gap between religion and disability by welcoming students from diverse backgrounds, including those with disabilities. Central to this transformation is the role of *kyais*, who preserve religious traditions as educators and spiritual leaders while guiding adaptations that foster empathy, cooperation and inclusion. Their leadership ensures pesantrens remain both relevant and responsive to contemporary needs. This commitment is reflected in various accommodations, such as accessible facilities, adaptive teaching methods and supportive technologies, that enable full participation of PWD in educational and religious activities. Through these efforts, pesantrens not only enhance the learning experiences of PWD but also promote broader values of social cohesion and mutual respect.
